# Effective health care for older people living and dying in care homes: a realist review

**DOI:** 10.1186/s12913-016-1493-4

**Published:** 2016-07-16

**Authors:** Claire Goodman, Tom Dening, Adam L. Gordon, Susan L. Davies, Julienne Meyer, Finbarr C. Martin, John R. F. Gladman, Clive Bowman, Christina Victor, Melanie Handley, Heather Gage, Steve Iliffe, Maria Zubair

**Affiliations:** Centre for Research in Primary and Community Care, University of Hertfordshire, Hatfield, AL10 9AB UK; Institute of Mental Health, University of Nottingham, Nottingham, UK; Division of Rehabilitation and Ageing, University of Nottingham, Nottingham, UK; School of Health Sciences, City University, London, UK; Division of Health and Social Care Research, Kings College London, London, UK; Department of Community Health Nursing and Health Studies, Brunel University, Uxbridge, UK; School of Economics, University of Surrey, Guildford, UK; Department of Primary Care & Population Health, University College London, London, UK; School of Sociology and Social Policy & Centre for Dementia, Institute of Mental Health, University of Nottingham, Nottingham, UK

**Keywords:** Realist review, Care home, Older people, Health outcomes, Long-term care

## Abstract

**Background:**

Care home residents in England have variable access to health care services. There is currently no coherent policy or consensus about the best arrangements to meet these needs. The purpose of this review was to explore the evidence for how different service delivery models for care home residents support and/or improve wellbeing and health-related outcomes in older people living and dying in care homes.

**Methods:**

We conceptualised models of health care provision to care homes as complex interventions. We used a realist review approach to develop a preliminary understanding of what supported good health care provision to care homes. We completed a scoping of the literature and interviewed National Health Service and Local Authority commissioners, providers of services to care homes, representatives from the Regulator, care home managers, residents and their families. We used these data to develop theoretical propositions to be tested in the literature to explain why an intervention may be effective in some situations and not others. We searched electronic databases and related grey literature. Finally the findings were reviewed with an external advisory group.

**Results:**

Strategies that support and sustain relational working between care home staff and visiting health care professionals explained the observed differences in how health care interventions were accepted and embedded into care home practice. Actions that encouraged visiting health care professionals and care home staff jointly to identify, plan and implement care home appropriate protocols for care, when supported by ongoing facilitation from visiting clinicians, were important. Contextual factors such as financial incentives or sanctions, agreed protocols, clinical expertise and structured approaches to assessment and care planning could support relational working to occur, but of themselves appeared insufficient to achieve change.

**Conclusion:**

How relational working is structured between health and care home staff is key to whether health service interventions achieve health related outcomes for residents and their respective organisations. The belief that either paying clinicians to do more in care homes and/or investing in training of care home staff is sufficient for better outcomes was not supported.

## Background

In the UK there are over 450,000 places in care homes catering for older people and those with physical disabilities [1]. A care home can offer personal care and 24 h support (often described as a residential home), with onsite nursing (often described as a nursing home), or a mix of the two. Care home residents have a high prevalence of functional dependency, cognitive impairment, multi-morbidity, frailty, polypharmacy, and behavioural symptoms [2, 3]. These characteristics result in complex healthcare needs. There is currently no national (England) policy or consensus about the best arrangements to meet these needs. Consistently, studies and professional reports have found that residents in care homes have inequitable access to components of health care that would be available to them from the National Health Service (NHS) [4–9] if they lived at home.

In response to such findings, or due to locally perceived problems, a wide variety of local health care delivery models have been developed. These range from the creation of care home specialist teams to the use of incentives and target-based models to change the behaviour of existing care providers. Some of these initiatives have been evaluated, usually in relation to the impact on the specific problem identified locally. Despite their promise, innovative approaches to health care delivery in care homes usually remain highly localised and are often short-lived. Implementing these models at scale and sustaining them beyond initial trial periods has proved difficult [10]. It remains unclear how best to spread successful approaches into routine practice [6, 11].

Interventions that provide health care to care homes do not work in and of themselves; they only have effects through the reasoning and reactions of their recipients [12]. To deal with this degree of complexity requires an appropriate methodology, such as realist review. Realist review is a theory-driven approach, which brings together evidence from multiple sources to make sense of complex interventions as applied in a range of situations and settings. It aims to build plausible and evidence-based explanations for the observed outcomes of complex interventions that have multiple components and involve multiple participants [13]. This realist review seeks to understand more about how key elements of health care provision to care homes work in different contexts.

### Objectives

The principal aim of this review was to develop a theoretical understanding of what features of different approaches to service delivery are necessary to improve health outcomes for care home residents.

The objectives for the realist review were to:

1. Develop an understanding of the key mechanisms by which different models of health care delivery attempt to achieve improved outcomes for care home residents.
2. Identify the characteristics of different models of health care delivery which were associated with positive impact on five key outcomes for NHS commissioners and service providers (namely: medication use; use of out-of-hours services; hospital admissions including emergency department attendances; length of hospital stay; and user satisfaction).

## Methods

The review draws on a realist approach [14, 15]. A more detailed description of the protocol and search terms used is published elsewhere [16]. The care home sector supports a population with common characteristics (e.g. age, cognitive capacity, life expectancy) but is characterised by heterogeneity in terms of its size, funding, workforce and workplace culture. A realist review approach can address this intrinsic variability and complexity and make sense of the various possibilities, inhibitors and unanticipated consequences that may influence the success of different approaches of health care provision to care homes. Our realist review employed an iterative three-stage approach. Stage 1 involved scoping searches and stakeholder interviews to identify sources of policy, legislative and professional thinking that could help explain how health care services and care homes work with each other. Stage 2 was a more in-depth review of the evidence to interrogate our explanatory model. Stage 3 used iterative searching to refine and revise how the candidate theories could be applied to the UK care home setting.

### Stage 1: Defining the scope of the review - concept mining and theory development

Stage 1 sought to establish the range of approaches used by NHS services to support health care for older people in care homes and to identify the implicit and/or explicit beliefs held by stakeholders which informed these various approaches in regard to what worked well or did not work. This research included: a preliminary scoping review of the literature on health care service provision to care homes; a survey of surveys of health care services provided to care homes; a review of reviews on care home interventions; and interviews with key informants involved in the commissioning, provision and regulation of health care to care homes, as well as recipients of care (residents and relatives). The interview element of the study was reviewed and supported by the University of Hertfordshire Ethics Committee reference: NMSCC/12/12/2/A. The detailed methods and findings from the survey of surveys and the stakeholder interviews have been published elsewhere [4, 16]. For the purposes of driving an ongoing iterative review, these findings were organised into summary tables that set out the range of service provision to care homes and the explicit and implicit assumptions about how effective working between health care services and care homes is achieved in relation to the pre-identified outcomes.

### Stage 2: Theory refinement and testing

**Fig. 1:**
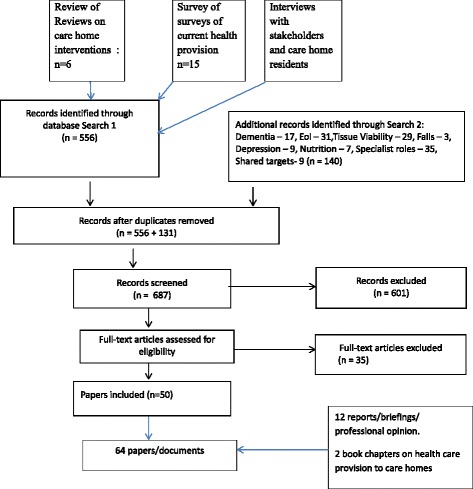
Flow chart of evidence retrieval

Stage 2 tested the relevance and rigour of emerging findings from Stage 1. Using the tabulated findings from this stage, the research team developed a series of statements that captured the emergent programme theories of how health care services worked with care homes. This resulted in a series of statements about possible context, mechanism and outcome configurations [17]. These informed how the academic and professional/practice based literature was identified and data extracted. More detailed searches of the literature then revisited and expanded the searches from Stage 1 and considered interventions that drew on theories that focused on: the assessment of frail older people in the last years of life [3, 18, 19]; system driven quality improvement schemes in primary care [20]; and theories of integrated working that emphasise relational, participatory, and context sensitive approaches in care home settings [21, 22] (Tables 1, 2 and Fig. 1).

**Table 1:** Preliminary Programme theories developed from Stage 1

Health care for older people resident in care homes achieves optimal outcomes if	How expressed in service delivery models/intervention research
**System based quality improvement approaches** incentivise health care staff (GPs and care home staff) regularly to visit and review residents’ health status then care home staff will prioritise the aspects of care activities that are being monitored, review of patient care and avoid inappropriate and avoidable use of urgent and emergency services	Interventions that use financial payments, sanctions and audit to improve particular health care outcomes and adherence to protocols and guidance
**Age-appropriate care** can be accessed by older people resident in long term care. Then residents will not have to wait to have symptoms treated and then they will experience fewer episodes of avoidable ill health	Interventions that focus on assessment maintenance and improvement of function, management of diseases and symptoms associated with old age through education, training of care home staff and access to visiting clinical experts and care home specialist teams
Interventions are predicated on **establishing relational approaches** that promote integrated working between visiting health care and care home staff. Staff will become less risk averse, trust each other’s opinions and be willing to engage with activities that promote residents’ health and support them to stay in the care home.	Emphasis on strategies that support co-design and joint priority setting to achieve improved outcomes for residents, e.g. shared education and training, continuity of contact with particular clinical experts, shared learning, feedback on achievements between health and care home staff

bold type denotes the working title of each programme theory

**Table 2:** Focus of care home papers reviewed

Research focus of papers reviewed with one or more outcomes of interest (medication use; use of out-of-hours services; hospital admissions including emergency department attendances; length of h*o*spital stay; and user satisfaction)	References
Medication management	[31–33, 39, 84–90]
End of life care	[49, 55, 56, 62, 90–94]
Resident health promotion (e.g. nutrition, flu prevention, tissue viability, oral health, functional improvement, dementia care, falls prevention)	[48–50, 53, 58, 60, 61, 74, 95–98]
Management of depression and related interventions	[36, 39, 45, 46, 60, 61, 85, 99–101]
Pay for performance/audit	[28, 30, 101, 102]
Interventions to promote health service use, integration of health and social care services in care homes including specialist roles and reduce use of secondary care	[11, 13, 27, 38, 65, 70, 102–106]

In keeping with realist inquiry methods, equal consideration was given to negative and positive outcomes and inconsistencies in accounts of what works, when and with what outcomes.

Relevant literature was requested through primary care and care home networks comprising: the *My*HomeLife Network, National Care Home Research and Development Forum, Dementia and Neurodegenerative Diseases Research Network (DeNDRoN), Clinical Research Networks and care home provider organisations and associations including Care England and the National Care Forum and the Residents and Relatives Association.

The following electronic databases were searched: Pubmed, CINAHL (Cumulative Index to Nursing & Allied Health Literature), The Cochrane Library (including the Cochrane Database of Systematic Reviews), DARE (Database of Abstracts of Reviews of Effects), the HTA Database, NHS EED (NHS Economic Evaluation Database), Scopus, SocAbs (Sociological Abstracts), ASSIA (Applied Social Sciences Abstract & Indexes), BiblioMap (The EPPI-Centre register of health promotion and public health research), Sirius, OpenGrey, Social Care Online, the National Research Register Archive, the National Institute of Health Research portfolio database, Google and Google Scholar. Search terms used were organised to capture the range of possible interventions that health care services provide (e.g. falls prevention, wound care, end of life care) and included studies that focused on achieving change in one or more of the outcomes of interest (for example prevention of hospital admission, resident satisfaction). These searches were complemented by lateral searches of reference lists from primary studies and relevant systematic reviews.

Initial searches excluded publications prior to 2006, because the later period has seen social care and independent care providers take responsibility for long term care of older people. It has also coincided with a rapid growth in care home research outputs [23].

We included studies of any research design, unpublished and grey literature, policy documents and information reported in specialist conferences.

Studies reviewed had to be relevant to UK systems of health care. We treated with caution or excluded studies where the care home medical support would be in-house (as in the Netherlands) or the level of care would be closer to hospital level provision (as can be the case in the US). Relevant studies therefore described health care provision to care homes that were similar or could be applied to UK working patterns. Included studies focused on health care to care homes provided by visiting health care professionals or services. Studies that were not UK-based and where there was transferable learning were included. These tended to be those that had been identified from iterative searches and that reinforced or challenged something identified in the UK literature.

Four reviewers (CG, SLD, MZ, MH) independently screened titles and abstracts to identify potentially relevant documents, which were then retrieved and assessed according to one or more of the following inclusion criteria:

- Studies which considered residents in a care home with specific health needs/problems and focused on one or more of the outcomes of interest
- Studies of any intervention designed to improve the health status of care home residents that involved visiting health care professionals and offered opportunities for transferable learning to a UK setting
- Studies that provided context relevant evidence on the implementation and uptake of interventions in care homes generally (not confined to health care), that also helped build our programme theories and logic.

Data extraction proceeded as follows. In order to identify key elements of importance to the success or failure of an intervention or models of service delivery in particular contexts, information was gathered on how health care was organised, funded, provided and delivered. Particular attention was given to the detail or thick description of the process, how the underlying assumptions and theoretical framework (if identified) were articulated, and whether this fitted with the focus of our review in terms of the underlying theory and the impact of the intervention on the outcomes of interest. Our approach drew on Rycroft-Malone et al.’s [24] approach to data extraction in realist synthesis that questions the integrity of each theory, considers the competing theories as explanations to why certain outcomes are achieved in similar and different settings and compares the stated theory with observed practice.

### Stage 3: Analysis and synthesis processes

A realist analysis of data adheres to a generative explanation for causation and looks for recurrent patterns of outcomes and their associated mechanisms and contexts (CMO: Context-Mechanism-Outcome configurations) that occur in the evidence reviewed [13]. Data synthesis was led by CG with ongoing face-to-face and virtual discussions with SLD, MZ, MH and the wider research team. As the review progressed, discussion focussed on particular papers and sources that offered competing accounts of why or how an intervention was chosen and why it had, or had not, worked. We concentrated on what appeared to be recurrent patterns of contexts and outcomes in the data (demi-regularities) and then sought to explain these through the means (mechanisms) by which they occurred.

It was frequently stated, for example, in policy documents, professional literature and recommendations from descriptive studies that the alignment of general practitioners to specific care homes (for example by having all residents registered with one GP Practice) was important in improving health outcomes for care home residents. For data extraction purposes, any descriptions of the way that the GP/GPs worked or the detailed arrangements for access or reviews etc. were scrutinized in terms of alignment with the provisional programme theories. Thus, in studies where the GP was the main access to and provider of health care, we considered whether any specific elements or mechanisms within the service (e.g. the use of payments, GP knowledge, the frequency of contact, or a particular way of working with care home staff) could explain why and in what contexts our outcomes of interest were achieved (or not).

The review’s preliminary findings were presented to the study advisory group for further discussion and challenge. The study advisory group was an invited body of nine members, including commissioners, practitioners, care home owners and older people representatives, all with expertise in providing or receiving health care services to care homes. This iterative discussion process compared the stated theory with actual practice. In particular, we considered which aspects of different interventions comprised mechanisms by which delivery of appropriate health care to care homes might be realised and which aspects comprised contexts which supported or inhibited the identified mechanisms.

## Results

Fundamental to realist approaches is the identification and refinement of a series of propositions about how a programme might work to achieve its outcomes [25]. Stage 1 identified six potential candidate programme theories (later refined to three) of how health care services to care homes improved the health of residents and use of services. These considered that health outcomes for care home residents could be improved when:

1. Tailored education and support for care home staff provided by clinical experts and supported by the use of structured documentation and protocols will improve resident outcomes by prioritising specific assessment/care activities that trigger changes in how residents’ care is planned and how care home staff recognise and frame their need for training and support from visiting clinicians
2. Contracting and financial incentives paid to doctors (GPs) to provide dedicated services to care homes, with audit against pre-specified process and outcome measures will change the pattern and frequency of GP contact with residents and staff, increase the time and opportunities for screening and review of care, increase staff confidence that they can access a GP and reduce demand on emergency and secondary care services
3. Formalised recognition and ongoing facilitated support of care home staff to equip them to build relationships and work with health service providers will validate the expertise of care home staff, increase their confidence when working with visiting health care professionals, and lead to care home staff identifying priorities for residents’ health care with visiting health care professionals
4. Appointment of care home champions with specialist expertise in quality of care for older people and designated responsibility to work with care homes will provide expertise and continuity of support to the care home staff encourage skills acquisition that would cause staff to be more proactive in providing health care to residents
5. Commissioning and provision of services that focus on specific problems and health care needs frequently experienced by care home residents (for example, falls prevention, end-of-life care, continence management) changes the focus from care home residents to the individual needs of patients and triggers a service response that is equivalent to that received by people living in their own home
6. Building of inter-organisational and inter-sector networks (health/social/public/private sectors) will change how different services work together to highlight gaps and overlaps in service provision, this will trigger conversations and planning between services about resource use and who is responsible for providing health care.

In discussion with the advisory group, the research team focused on areas of overlap and fit with the preliminary scoping of the literature and review of service provision and likely configurations of enablers and barriers that might shape how these interventions work. This resulted in three broad programme areas that were the basis for the second stage of the synthesis, and focused on care home-specific evidence on ageing and frailty, system change and cross organisational working between care home and visiting health care staff (Table 1). These programme theories are each discussed in turn below.

The searches (Fig. 1) initially considered care home wide interventions and then topic led interventions that were linked to one or more of the five outcomes listed in Objective 2 (see above). This generated 687 records. Following screening and de-duplication, 86 full text articles were assessed for eligibility and 64 were included; 53 of which reported on at least one of the outcomes of interest. Papers were excluded for the following reasons: not care home specific; either did not include the outcomes of interest or provided no or insufficient detail about health care provision to care homes; or could not be linked to UK systems [26].

### System based quality improvement mechanisms to improve health care outcomes: the use of incentives, sanctions and targets

The theoretical basis for the use of system-based incentives, targets and sanctions is that they prompt behavioural change through targeting particular professional groups or organisations (in this case, care homes), focus on the improvement of specific processes or outcomes, and thereby improve quality of care and reduce inequity of provision [27]. The Quality Outcomes Framework (QOF), introduced for GPs in England in 2003, linked financial incentives to the quality of care that is provided by practices [28] and has been described as a lever to reduce health inequalities and reinforce evidence based practice [29].

Possible Context-Mechanism-Outcome configurations based on the different theoretical propositions were tested through the literature review, see below, (words in italics emphasise the suggested mechanisms). It makes explicit the differentiation between how we understood the intervention or the allocated resources used to provide care and the possible mechanisms that are the possible trigger for change (Context *Intervention* Mechanism Outcome).

The following possible C(I)MO configuration to explain how incentives and sanctions paid to primary care can improve health care in care homes were identified:

- **Context:** Care home staff have intermittent contact with the residents’ GP; encounters with primary care are usually unplanned and in response to an urgent need and this affects the proactive identification of residents’ health care needs, access to and quality of care and frequency of acute episodes of ill health.
- **Resources/Intervention:** GPs are provided with a range of incentives and sanctions to visit regularly and undertake resident assessments in key areas of care for example medication review, and provide the care home with support and advice in addition to individual patient visits.
- **Mechanisms:** GPs are **motivated** to engage with the care home staff because of the incentives and sanctions that **prompt** them to complete regular reviews of care home residents and ***work with care home staff*** to plan care **and*identify*** residents in need of additional support and care.
- **Outcomes:** Care home staff are more confident working with GPs around particular areas of care, specifically medication management and reduced use of OOH and emergency services.

For General Practitioners (GPs) working with care homes, rewards linked to particular clinical activities are used as incentives to define and increase the length and frequency of their visits in order to achieve the desired outcomes of continuity of contact and proactive approaches to patient care [30]. A focus of the evidence reviewed about the use of incentives and sanctions was around its role in the improvement of medication management [31]. This literature suggests that additional payments to GPs and pharmacists to do specific activities can improve monitoring of medication use. However, the use of payments or sanctions alone to trigger GP involvement in resident assessment and review did not appear from the evidence reviewed to be sufficient to improve activities such as regular medication review, prescribing and related resident outcomes. Three further factors were also identified: the need for an accountability structure, named professionals used to deliver a specified intervention, and care home-sensitive protocols which took account of the high prevalence of dementia [32, 33]. Other contextual factors included the need to consider those residents at particular risk and also care home staff’s need for ongoing support and training.

Generally, the literature would appear to support the view that, whilst incentives can improve the process of care and productivity (for example, better adherence to protocols and care pathways), the evidence of their impact on patient outcomes is limited [34, 35]. Charlesworth and colleagues argued that:

“*Incentive schemes can only work if the organisations and clinicians whose behaviour they are trying to change****understand what is required (****our emphasis****)****. Too often, the incentives are blurred or inconsistent. In part, this is a result of the complexity of the current system” p14*

The main pay-for-performance approach in UK primary care (Quality Outcomes Framework) allows practices to exclude patients for reasons such as extreme frailty, or evidence of decline. This arguably creates an implicit expectation that payment is linked to a set of outcomes that are less relevant or irrelevant to care homes [36]. Residents with dementia achieve lower quality indicators in the QOF pay-for-performance system than their community dwelling counterparts [36]. Indicators focus on very specific aspects of disease management. Care home residents as a discrete population may not be recognised by GPs as a priority group in need of identification and active management. Payments alone may not be sufficient to change that view, make care home residents a GP priority or address issues of accessibility, appropriateness or system co-ordination [37]. As Roland, observed when commenting on the evidence for pay for performance for GPs:

*They (incentives) work best when all the ducks are lined up in a row: financial, organisational, and professional incentives, then the incentives are providing****encouragement****(our emphasis) to do the things that doctors believe they should be doing anyway* (Martin Roland When incentives go wrong http://www.cchsr.iph.cam.ac.uk/2107).

One small study audited cases of residents’ admission to hospital as a trigger to identify and discuss with GPs the factors influencing hospital admissions from care homes. The authors reported a change in GP behaviour, with an increase in care home visit rates and a reduction in overall hospital admissions [38] but the audit and review had no impact on the numbers of hospital admissions initiated by care home staff. The authors suggested that care home staff, particularly where there was no on-site nursing provision, needed further support from visiting health care professionals and involvement in anticipatory planning for residents at risk of hospital admission.

The Evans et al. (2010) study was the only study we found that explored and reported how the mechanism of providing feedback on GP performance could influence how GPs worked with care homes. Other studies suggested that formal notification to GPs of the need to improve care or guidance on good practice (prescribing), did not provoke change [39, 40]. A possible explanation is that feedback on medication management does not have the same impact as alerts about unplanned hospital admissions that are recognised as avoidable and costly. This suggests that it is the urgency of the issue to the health service, as opposed to its impact on individual residents or care home staff that influences when audit and feedback mechanisms trigger increased engagement with care homes by NHS services.

We found no evidence that targeted payments prompted an increase in health care professional visits or assessment of care home residents’ health care and medication needs. One US study found that financial payments, when paid directly to care homes as opposed to visiting health care professionals, improved resident outcomes but this was for specific projects identified by care home staff. The incentive was to introduce new approaches to care, not to ensure that health care was provided [28].

### Age appropriate care can be accessed by older people resident in long term care

There is evidence that systematic approaches to the assessment and management of older people can reduce mortality and improve function [41–43]. These interventions rely on the involvement of clinicians with expertise in the care of frail older people and their ability to work with others to implement care plans. Box 2 illustrates a C(I)MO proposition outlining how services that focus on providing expertise in age appropriate care could work.

The following possible C(I)MO configuration explains how provision of expert practitioners in old age care can improve health care in care homes:

- **Context:** Care homes have unpredictable access to health care services, the majority of staff are not clinically qualified, residents are frail and in the last years of life with complex health and social care needs.
- **Resources/Intervention:** Experts in care of older people visit care homes regularly to compensate for known deficits in knowledge and skills.
- **Mechanisms:** Care homes staff feel ***supported*and*trained*** in how to provide care to frail older people. They are ***motivated*** to learn new skills because of the facilitation and ongoing expert support they receive.
- **Outcomes:** Care home staff are more confident and skilled in looking after care home residents and specific areas of care. Residents' function is improved or maintained and staff have higher levels of job satisfaction and the care homes are less likely to use emergency and out of hours services,

An increasing body of work has developed interventions for care home residents that have focused on specific processes such as assessment, targeted interventions and protocol-based care. Examples include comprehensive assessment [44], depression [45–47], dementia [7], falls prevention [48], nutrition [49–52], recovery from stroke [53], medication [54], end of life care [55–57], tissue viability [58], oral hygiene [59], and occupational therapy interventions [60, 61]. Most of these interventions were multicomponent but had in common the detailed assessment of residents’ functional abilities and the teaching of new skills to care home staff to improve residents’ health and wellbeing.

Most but not all interventions were appreciated by care home staff, often with reports of increased staff confidence that could have acted as a feedback loop and potential additional mechanism to influence improved residents’ health. However, the positive response of staff was as likely to have been a reflection of care home staff’s previously limited experience of professional support and encouragement. Where there was a comparative study, the control was invariably usual care or provision of written materials. This suggests that the mechanism that triggered a change in staff (or not) was the process of working together and receiving clinical support. The underlying assumption of many of the studies reviewed, that the allocation of professional (biomedical) expertise, education and training of staff and identification of people at risk would lead to improved health outcomes, was not supported.

These were important contextual factors necessary for change. They were not, however, the mechanisms that provided the generative force to achieve the resident outcomes.

Several contextual factors have been suggested that may inhibit care homes and/or residents ability to engage with interventions, but these remain largely untested. Putative factors include care home size and ownership, staff turnover, percentage of residents who have been resident in the care home for less than 12 months, and the absence of additional triggers or mechanisms such as the involvement of care home leadership, staff qualifications and the duration of programmes [47]. Two studies on end-of-life training programmes found that use of advanced care planning documentation, improved staff satisfaction and reduced hospital deaths were positively associated with how long the care home manager had been in place, prior training in end of life care and low staff turnover [55, 62].

One study with a positive outcome appears to have been successful because of particular contextual factors. Researchers [63] tested the effectiveness of an influenza vaccine programme for care home staff (not residents) to prevent death, morbidity and health service use. The mechanisms of interest within the programme were the identification of a key link-worker within the care home and the development of tailored processes to encourage vaccination uptake by care home staff. These were supported by a care home policy for immunisation. It achieved significantly lower mortality of residents in intervention homes compared with control homes. The key differences between the intervention process described in this study and that of the others reviewed was that it was a single, time-specific intervention that could be co-ordinated by one member of staff per care home. It was, in comparison to the other studies, a simple intervention with a quantifiable outcome where the proposed health benefits to both staff and residents were clear for staff and residents [64]. An expert practitioner was important as a resource that enabled the link worker in the care home to implement the immunisation process that generated the positive outcomes.

### Relational approaches to promote integrated working between visiting health care and care home staff that emphasise interpersonal skills and shared decision making

The competing priorities of health and social care staff, inherent power imbalances between qualified and unqualified staff, staff turnover and the difficulties health care professionals have in understanding the predominantly private care home environment are well documented barriers to effective collaboration between health and care home staff [50, 64–67]. Relational working draws on theories that emphasise strategies that coordinate and support shared problem solving (and not blaming) and working relationships that are grounded in common goals, shared knowledge and mutual respect [22, 68, 69]. In the extraction of data in this stage of the review (based on the stakeholder interviews and the preliminary scoping) relational working was characterised as those activities and processes which emphasise shared-decision making, planning and learning and continuity of contact between staff from different sectors.

The following possible C(I)MO configuration explains how an intervention designed to improve relational working achieve improved outcomes for care home residents and staff involved:

- **Context:** The expertise of care home staff in providing care for older people with frailty and/or dementia is seldom recognised by visiting health care professionals. Health care interventions, emphasising physical health, do not fit well with care home priorities of providing a homely setting and working practices that seek to balance positive risk taking with patient safety. Working patterns to facilitate in reach from numerous health professionals are difficult to accommodate by care home staff with limited resources who want to achieve a more personalised environment for residents.
- **Resources/Intervention:** Models of care that introduce opportunities for joint priority setting and processes that support ongoing discussion and review of residents’ health care needs between care home and visiting health care professionals.
- **Mechanisms:** Identification of key personnel in the care home to work with visiting health care professionals trigger a **response** where staff are motivated to develop shared priorities for care and **a sense of common purpose** because their views are valued, they develop approaches that **fit** with the care home working patterns, **incorporate** care home staff knowledge and priorities **are jointly** agreed, enacted and reviewed.
- **Outcomes:** Care home staff and visiting health care professionals are motivated to work together and improve care for residents in agreed areas of practice. Residents’ function is improved or maintained; staff have higher levels of job satisfaction; and the care homes are less likely to use emergency and out of hours services.

The organisation of care between the resident, their relatives, care home staff and visiting health care professionals requires more than the one-on-one encounter between clinician and patient. It is a negotiated process within a changing environment. Over time, there may be individual and organisational changes in who has responsibility for providing and/or paying for care, and changes in the arrangements for commissioning health and social elements of care. Roles and responsibilities for a resident’s care can shift as a consequence of an acute health event and/or a gradual shift in need from “social” to “health” care as complex long-term conditions progress and predominate, and/or as part of a transition to end-of-life care [70, 71]. These observations highlight the importance of understanding how the arrangements for health service involvement support, or inhibit, the development of networks of interprofessional collaboration and care, specifically how they impact on relational working. In the extraction of data in this stage of the review (based on the stakeholder interviews and the preliminary scoping) relational working was characterised as those activities and processes which emphasise shared-decision making, planning and learning, and continuity of contact between staff from different sectors.

Three contextual factors reflecting aspects of relational working were identified as important for triggering activities and processes that were likely to lead to improved outcomes. These were important whether the intervention being reported had an explicit focus on working with care homes collaboratively or not.

### Shared priority that fitted with care home workflow

Most of the health care interventions reviewed were multi-component. Interventions were more likely to have positive uptake and promising outcomes (completion of education and training programmes, improved documentation of residents’ care) where they focussed on a concern of mutual interest to care home and healthcare staff and/or residents and family. For example, end of life care that avoided unplanned hospital admissions and enabled the person to die in the care home fitted with care home staff views that they were the person’s proxy family, that the care home was the person’s home and that it was distressing to be with strangers (hospital staff) at the end of life [56, 72].

Where the initiative was identified as a priority based on a review of resident need, but not recognised by staff as such (particularly where it added to their workload), it was unlikely to be implemented or sustained [47, 73]. As one study that introduced a therapy-led intervention to reduce depression observed:

*At times it was difficult to explain our remit to staff. We had little time to change attitudes of some staff to issues of mobility; making it hard to facilitate a change in practice* Underwood et al. [47] (p 2013)

This relates to who the health care professionals worked with and their role in care delivery. Studies showed that interventions were more likely to be acceptable and effective when there was a nominated person in the care home to liaise with, particularly where this person could play a collaborative role in reviewing, planning and supporting care [63].

### Fit with the care home workplace

There was evidence of improved outcomes where care home staff had flexibility in how an intervention was implemented [39, 63, 74]. This was particularly the case where there was access to expert facilitation and support. Emphasis on preparatory work, structured assessment of a care home’s readiness to participate, collaborative and bottom-up approaches, shared learning and the development of a common understanding between care home staff and health care providers were key mechanisms for improvement and involvement of care home staff in the intervention [58, 67, 75]. In one study this involved developing an intervention with care home managers that built on previous staff learning in end of life care, it was an iterative and reflective process that involved day and night staff and sought to address care home specific issues such as supporting people with dementia:

“*We think this success (reduction in hospital deaths, improvement in quality of life for residents with dementia) is related to the training****addressing staff fears and problems****(our emphasis) as well as increasing knowledge” Livingston,* et al. *2013 (p1587*)

The involvement of care home staff, particularly senior staff, and other psychological and contextual factors that could be characterized collectively as a care home’s readiness for change positively impacted upon the uptake of innovation [50, 76].

Bamford and colleagues found that whilst some changes could be achieved in staff understanding of nutrition the implementation of nutrition guidelines in care homes foundered because:

*It proved difficult to build collective understanding of and commitment to the study resulting in inconsistent implementation…Managers’ commitment to the nutrition guidelines did not extend to using scarce resources to facilitate implementation (p10)*

This finding was resonant with multiple references in the reviewed texts to the probable influence of the leadership and culture of particular care homes on health care outcomes and staff satisfaction.

### Access to ongoing support and facilitation

We were unable to find any evidence to support the widely expressed belief that attachment of one GP practice per care home improved resident outcomes. In fact there was evidence that GP allocation did not lead automatically to continuity of support and could have the unintended consequences of rationing care because GPs set regular but fixed times for their availability [77]. There was also evidence that one practice per care home arrangements could effectively trap providers in dysfunctional relationships, providing an adverse context for appropriate health care delivery [70].

Ongoing support from a clinician or team with relevant expertise was nevertheless important, and especially so, was *how* this was delivered. Where the facilitator or lead clinician was able to be present and responsive to the needs of particular residents as they arose and engage staff in action learning that focused on issues of interest to staff, this was linked to higher levels of staff engagement and fidelity with training [11, 26, 39, 74, 78] when compared to interventions where the clinician input was episodic or task focused [54, 79]. The mechanism within the facilitation process was when the health care professional worked with staff as the “bridge” to connect between interventions to improve health care of residents over time in a way that could be modified or be incorporated into existing patterns of working.

## Discussion

This realist review has identified emergent patterns or *demi-regularities* in the evidence reviewed that underline the importance of *how* health care professionals introduce and provide health care support to care homes. The way in which they work with care home staff, residents and their families, and the duration of this relational working is important regardless of the specific health issue targeted.

Broad mechanisms within a programme that can help deliver appropriate health care to care home residents are those activities within an intervention that ensure the intervention is specific to the care home, aligns with the goals and priorities of care home staff, is not adapted from other care settings and patient groups, and that from the outset focuses on activities that aim to build relationships between care home staff and visiting health care professionals. The contextual elements that shape the achievement of these outcomes as well as sustain participation are many: care home readiness to work with health care staff (e.g. care home leadership and previous history of collaboration), availability of structured assessment and care plans, involvement of a health care professional to support change and reinforce learning and organisational endorsement, financial remuneration, and staff incentives.

This is consistent with what is known generally about integrated working and the barriers to horizontal and vertical integration across health and social care organisations [80]. From the evidence reviewed, the relevance and usefulness of the health care interventions and ultimately their impact were diminished in situations where there was either little evidence of prior collaborations, or failure to engage in a period of exploration and preparation that could shape how health care professionals and care home staff could work together. It highlights the levels at which care homes and NHS work have to work together-structural, service and personal, to achieve the desired outcomes.

Interventions alter context, that is, they attempt to change the care home environment so that the correct mechanisms are ‘triggered’ to generate the desired outcome. They do not in themselves have causal powers. Interventions that created the mechanism where staff felt they had a common purpose were necessary for change to occur. This was achieved by connecting new knowledge with existing practice and knowledge, using processes such as care planning and ongoing conversations to reconcile competing priorities in the care home. These findings resonate with international studies on the implementation of evidence-based care in residential care facilities and working with care home staff to improve residents’ well-being [81, 82]. A review on the use of advance care plans that included care homes [83] argued that no amount of facilitation or structured tools will reduce the effects of those things that undermine them. Interventions that were workable within time-pressured environments, whose mechanisms support dialogue, experimentation and collaboration, and allow the system to evolve and self-organise over time, were most likely to be effective.

These conclusions parallel those of this review. Interventions to deliver appropriate health care are more likely to be successful when their mechanisms accommodate and recognise the interactional nature of decision-making in care home settings and can adjust for competing work demands in the care homes. Mechanisms of successful programmes were characterised by activities that provided visiting health care professionals and care home staff allocated time for discussion, reflection and allowed reconfiguration of the intervention to match care home workflow and priorities. Contextual influences such as financial incentives or sanctions, agreed protocols, continuity of contact and evidence based approaches to assessment and care planning provided the necessary equipment or resources to enable those mechanisms to achieve improved resident and staff outcomes.

### Strengths, limitations and future research directions

The contribution of this review is that it has drawn together a disparate literature on care home residents’ access to health care. It has tested the key proposition (drawn from stakeholder interviews, current models of service delivery, and preliminary scoping) that those elements or mechanisms of interventions that address *how* practitioners work together are key to achieving changes in practice. The overall strength of evidence supporting the explanatory insights is constrained by the lack of detail of the processes at work in the various interventions and by a focus on staff satisfaction and confidence, rather than considering resident priorities, observed changes in practice and measurable changes in resident outcomes. We were unable to provide a more detailed analysis of how certain contexts generated the mechanisms identified.

A strength of the realist synthesis process is that we were able to develop and test a theoretical understanding of what supports health care provision to care homes. We tested and debated the relevance and resonance of the emergent findings with stakeholders through interviews, presentations and meetings. Previous review and survey work has demonstrated the complexity of the setting, the paucity of evidence and the shortcomings and inadequacies of either care home providers or health care providers [4, 70]. In realist terms, even when the desired outcomes are not achieved there is opportunity to learn from the evidence and develop a theoretical understanding of what needs to be in place. This review helps to explain, in part, why intervention studies designed to improve care home residents health are unsuccessful.

As we have mentioned earlier, we have focused on situations where most health care for care homes is provided by external health care professionals and services, and our stakeholder interviews were limited to the UK. Nonetheless, our findings can be generalised to other countries where similar arrangements pertain, so this review is not solely relevant to the UK.

The findings from this review lay down a challenge to commissioners and providers of health care services to care homes. Interventions that do not include priority setting and service design as a shared enterprise between care home staff and health care practitioners, that fail to tailor practices to fit with care home working, or don’t provide ongoing support to staff will probably have limited impact on residents’ health and use of hospital services. The belief that either paying clinicians to do more in care homes and/or investment in training of care home staff is sufficient to improve outcomes was not supported. Any programme under development should incorporate practical outcome and experience measures into its design.

## Conclusions

This review provides an emergent conceptual model to understand statutory health care provision to UK care homes that has “practical adequacy”, that is to say, it has articulated what are likely to be the minimum requirements when providing a health care service to care homes. It has done this by setting out the evidence for the different possible context-mechanism-outcome configurations that need to be in place for health care delivery to the sector. More importantly, it has argued that interventions are more likely to achieve the outcomes of interest when they trigger the engagement of care home staff from the outset, and the intervention is structured to fit with the priorities and working practices of the care home. This has implications for how future research on health care in care homes should be designed and conducted.

## Abbreviations

C(I)MO, context (intervention) mechanism outcome; GP, general practitioner; NHS, national health service; QOF, quality outcomes framework.
